# Prevalence of Oral Pathogen *Slackia exigua* among Clinical Orthodontic and Non-Orthodontic Saliva Samples

**DOI:** 10.3390/microorganisms11040867

**Published:** 2023-03-28

**Authors:** Ching Shen, Justin Simpson, James Brigham Clawson, Steven Lam, Karl Kingsley

**Affiliations:** 1 Department of Advanced Education in Orthodontic Dentistry, School of Dental Medicine, University of Nevada-Las Vegas, 1700 W. Charleston Boulevard, Las Vegas, NV 89106, USA; 2 Department of Clinical Sciences, School of Dental Medicine, University of Nevada-Las Vegas, 1700 W. Charleston Boulevard, Las Vegas, NV 89106, USA; 3 Department of Biomedical Sciences, School of Dental Medicine, University of Nevada-Las Vegas, 1001 Shadow Lane, Las Vegas, NV 89106, USA

**Keywords:** *Slackia exigua*, saliva screening, oral prevalence

## Abstract

*Slackia exigua* (SE) is a recently identified intestinal microbe, which recent oral surveys suggest may be associated with oral diseases including caries and periodontal disease. Based upon the lack of information regarding this organism, the primary objective of this study was to determine the oral prevalence of this microbe and any potential associations with patient characteristics such as age, sex, or the presence of orthodontic appliances. This retrospective study involved the screening of an existing saliva repository composed of previously collected unstimulated clinical saliva samples. More specifically, N = 266 were identified and screened using a spectrophotometer at absorbances of A260 and A280 nm to determine their DNA purity and concentration. qPCR screening of these samples revealed a higher prevalence of *Slackia exigua* positive samples among pediatric patients (63.1%) compared with adults (36.9%) in this clinic population, *p* = 0.0007. In addition, higher percentages of *Slackia exigua* were observed among orthodontic patients (71.2%) compared with non-orthodontic patients (28.8%), *p* = 0.0001. These results did not vary by sex with nearly equal percentages of *Slackia exigua* positive males and females among adult and pediatric patients, as well as orthodontic and non-orthodontic samples. These results suggest a strong potential for association between the prevalence of this organism with age as well as orthodontic status, given that younger patients and those with orthodontic brackets (regardless of age) were most likely to harbor this pathogen in sufficient levels to be detected in saliva. More research will be needed to determine any associations with specific outcomes, such as caries or periodontal disease, among *Slackia exigua* positive patients within these specific populations.

## 1. Introduction

*Slackia exigua* (SE) was originally identified as a novel pathogen in oral lesions as *Eubacterium exiguum* [1]. This organism was reclassified as *Slackia exigua* based upon extensive analysis of 16S rRNA sequence comparisons and other phenotypic classification criteria [2]. The phylogenetic relationships between Slackia and Eubacterium species have been further delineated and refined, with additional related members now separately identified as *Eggerthella lenta* and *Mogibacterium* spp. [3,4].

As an obligate anaerobe, the gram-positive bacillus *Slackia exigua* has been identified among intestinal microbial communities, and was identified as a potential pathogen in abscess formations originating from the intestinal tract [5,6]. These studies have also revealed that *Slackia exigua* is not only a gram-positive strict anaerobe, but an organism that is non-motile, non-spore forming, and asaccharolytic [5,6] Moreover, this organism is considered fastidious or difficult to culture under laboratory conditions (requiring Anaerobic Blood Agar containing Vitamin K, hemin, and L-cysteine) and has been found unreactive in most conventional biochemistry tests, including urease production [2,6]. More recent research has identified *Slackia exigua* among patients with complex and multi organism diseases, such as severe early childhood caries (SECC), as well as persistent apical periodontitis [7,8]. In fact, this organism has been found as an early secondary colonizer in dental biofilms in vivo [9].

Since these discoveries, *Slackia exigua* has been demonstrated to function in the pathologic development and progression of both periodontal and cariogenic oral diseases [10,11]. Although two previous studies have found associations with this organism among smokers and patients with cognitive dysfunction, less is known about the oral prevalence of this organism among other patient populations [12,13]. Due to the known associations with caries development and fixed orthodontic appliances and treatment, the prevalence of this organism may be higher among this specific patient population—although no studies to date have evaluated this potential relationship yet [14,15].

In addition, due to the known association between fixed orthodontic appliances and both gingivitis and periodontal disease, these associations may also potentially influence the oral prevalence of *Slackia exigua* [16,17]. Based upon the lack of information regarding the prevalence of this organism among these patient populations, the primary objective of this study was to determine the oral prevalence of this microbe and any potential associations with patient characteristics such as age, sex, or the presence of fixed orthodontic appliances, such as brackets.

## 2. Materials and Methods

### 2.1. Study Approval

This study involved a retrospective analysis of clinical saliva samples from an existing biorepository within a public dental school. The protocol for this study was approved by the University of Nevada, Las Vegas (UNLV)—Institutional Review Board (IRB), as well as the Office for the Protection of Research Subjects (OPRS) as Research Exempt. The Protocol #1717625-1 “Retrospective analysis of microbial prevalence from DNA isolated from saliva samples originally obtained from the University of Nevada, Las Vegas (UNLV) School of Dental Medicine (SDM) pediatric and clinical population” was approved on 3 March 2021. 

### 2.2. Original Study Sample Collection Protocol

The collection of saliva from the original study was approved in 2013 under the UNLV -IRB and OPRS protocol OPRS#1305-4466M titled “The Prevalence of Oral Microbes in Saliva from the UNLV School of Dental Medicine Pediatric and Adult Clinical Population” for a period of five years (2013–2018). Briefly, study participants who agreed to voluntarily participate were asked to provide Informed Consent (over 18 years of age) or Pediatric Assent (if under 18 years of age). Patients were randomly recruited and saliva samples were collected at the beginning of patient visits.

More specifically, all clinical samples from the original protocol were collected at the start of a randomly chosen clinic session, prior to the beginning of any clinical treatment or protocol. To randomize the selection process for sample selection and clinical saliva collection, clinical session dates were chosen randomly using an algorithm that assigns a specific clinic session with a number (e.g., Monday 1 April, AM patients = 1, Monday 1 April, PM patient clinic = 2) with a set of randomly generated numbers to identify which specific collection dates and times would be used for study enrollment and sample collection. Clinical faculty and dental students on duty at the selected clinic date and time were then provided with the sample collection materials, including sets of sterile 50 mL polypropylene collection tubes, a set of randomly generated labels, and a basic intake information sheet that only allows for the collection of basic demographic information, such as the age of the patient, patient sex or gender, and the age of the patient at the date of the collection. The collection of each clinical sample was limited to a maximum of 5.0 mL of saliva collected in the individual sterile polypropylene tubes, which were then labeled using the previously mentioned randomized, non-duplicated numbers that were specifically generated to prevent the potential sharing of (or linkage with) any patient-identifying information. All samples were then transferred to a biomedical laboratory for long-term storage at −80 °C in a designated freezer.

### 2.3. DNA Isolation and Analysis

DNA extraction from clinical saliva samples was performed using phenol:chloroform extraction and the TRIzol isolation reagent from Fisher Scientific (Fair Lawn, NJ, USA). In brief, saliva samples were thawed and thoroughly mixed before placing equal volumes of saliva (500 μL) and TRIzol (500 μL) into a sterile microcentrifuge tube and triturated prior to the addition of 200 μL of chloroform. Each sample was then incubated on ice for ten minutes prior to centrifugation at 12,000 relative centrifugal force (RCF) for 15 min at 4 °C with an Eppendorf Microcentrifuge from Fisher Scientific (Fair Lawn, NJ, USA). Following centrifugation, the aqueous upper-phase was removed and placed into a new sterile microcentrifuge tube with an equal volume of isopropanol. Each sample was mixed to precipitate the DNA and then centrifuged for an additional ten minutes at 12,000× RCF to pellet the DNA. Isopropanol was removed and sample DNA pellets were washed with 100% ethanol and centrifuged again for five minutes at 12,000 RCF. Ethanol was removed and sample DNA was resuspended using nuclease-free distilled water. Sample DNA was subsequently screened for quality and quantity using absorbance readings at A260 nm and A280 nm and a NanoDrop 2000 Spectrophotometer from Fisher Scientific (Fair Lawn, NJ, USA).

### 2.4. qPCR Screening

Samples were screened for the presence of microbes using the QuantStudio Real-Time Polymerase Chain Reaction (PCR) system by Applied Biosciences (Waltham, MA, USA). Each reaction for qPCR screening contained SYBR Green qPCR Master Mix reagents obtained from ThermoFisher Scientific (Fair Lawn, NJ, USA), consisting of ABsolute SYBR Green, validated forward and reverse primers at 10 uM concentration, nuclease-free water, and 2.0 μL of sample DNA. Cycle specifications included 15 min at 95 °C to initiate enzyme activation with 40 cycles that included 15 s at 95 °C (denaturation), 30 s of annealing using each primer pair-specific temperature, and 30 s at 72 °C (final extension). Validated primer sets included:

Positive control, bacterial 16S rRNA primer set

Forward 16S rRNA primer: 5′-ACG CGT CGA CAG AGT TTG ATC CTG GCT-3′; 

Reverse 16S rRNA primer: 5′-GGG ACT ACC AGG GTA TCT AAT-3′; 

Slackia exigua primer set

*Slackia exigua* forward primer 5′TGC CTG CTG CAT GGT GGG TG-3′

*Slackia exigua* reverse primer 5′-AAA GGG ACA GGC CTG CTT C-3′

### 2.5. Statistical Analysis

Descriptive statistics of demographic (non-parametric) variables, including sex, age, and race or ethnicity were compiled and reported as percentages using Microsoft Excel (Redmond, WA, USA). Comparisons were made between the study sample and clinic population using Chi Square with a significance level set at alpha = 0.05. Following the screening of samples using qPCR, results were categorized as *Slackia exigua* (SE)-positive or *Slackia exigua* (SE)-negative for analysis using Chi Square statistics, which is appropriate for non-parametric (categorical data) analysis.

In addition, in order to determine the minimum appropriate sample size for this type of saliva-based microbial PCR screening, the recovery rate from the sample-limiting step of DNA extraction was used (90–95%) to establish the maximum expected experimental difference of 0.10 or 10%. Applying this with the significance level of alpha = 0.95 and a Power (p) of 0.90, a minimum sample size of N = 50 was calculated for this study.

## 3. Results

A total of N = 266 samples from an existing biorepository were identified for inclusion in this study (Table 1). In brief, nearly equal percentages of females (49.6%) and males (50.4%) were included in the study sample, which closely matches the overall percentages of females and males within the clinic population, *p* = 0.7518. In addition, the majority of samples were derived from minority (non-White) patients (65.1%), which was also similar to the percentage of minority patients within the overall clinic population (65.4%), *p* = 0.8422. The vast majority of these samples from minority patients were Hispanic (*n* = 151/173 or 87.3%). Finally, the average age of pediatric study samples was 13.1 years, which was higher than the average overall age of pediatric patients—mainly due to the study protocol inclusion criteria that specified patients aged seven years of age or older, *p* = 0.021. The age of adult patients averaged 40.6 years, which was younger than the overall age of adult patients within the clinic population of 42.3 years, *p* = 0.038.

DNA was isolated from each of the clinical patient samples and screened for concentration and purity (Table 2). These data revealed that the concentration of DNA from the pediatric patient samples averaged 481.2 ng/uL, ranging between 127.2 and 769.1 ng/uL. The average DNA purity as determined by the ratio of absorbance readings at A260 nm and A280 nm was 1.73, which ranged between 1.69 and 1.82. Similarly, the DNA concentration obtained from the adult patient samples was 455.3 ng/uL, which ranged between 189.5 and 776.2 ng/uL. The purity of DNA (A260:A280 ratio) averaged 1.75, ranging from 1.66 to 1.85.

**Figure 1:**
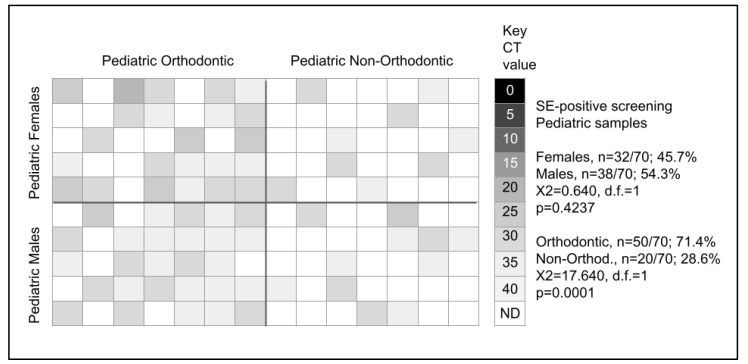
qPCR screening of pediatric samples for *Slackia exigua* (SE). Screening revealed *n* = 70/141 (49.6%) harbored *Slackia exigua* (SE), which was nearly equally divided among females (45.7%) and males (54.3%), *p* = 0.4237. The majority of *Slackia exigua* (SE)-positive samples were found among orthodontic patients (71.4%) versus non-orthodontic samples (28.6%), *p* = 0.0001.

Pediatric samples were then screened for the presence of *Slackia exigua* or SE (Figure 1). These data demonstrated that *n* = 70/141 or 49.6% of samples harbored DNA specific for this organism. Further evaluation of these data revealed that 32/70 or 45.7% of the *Slackia exigua* (SE)-positive samples were derived from pediatric females, with the remainder 38/70 or 54.3% from pediatric males, which was not statistically significant, *p* = 0.4237. However, the sorting of these data by orthodontic status revealed that *n* = 50/70 or 71.4% of the *Slackia exigua* (SE)-positive samples were found among pediatric orthodontic patients with only *n* = 20/70 or 28.6% from pediatric non-orthodontic samples, which was statistically significant, *p* = 0.0001.

More detailed analysis of pediatric female orthodontic patients revealed *n* = 23/35 or 65.7% were *Slackia exigua* (SE)-positive compared with *n* = 9/35 or 25.7% of pediatric female non-orthodontic patients, which was much greater than expected (X2 = 10.240, d.f. = 1, *p* = 0.0014). Similarly, analysis of the pediatric male orthodontic samples revealed *n* = 27/35 or 77.1% were *Slackia exigua* (SE)-positive compared with *n* = 11/36 or 30.6% of pediatric male non-orthodontic patients, which was much greater than expected (X2 = 29.160, d.f. = 1 *p* = 0.0001).

**Figure 2:**
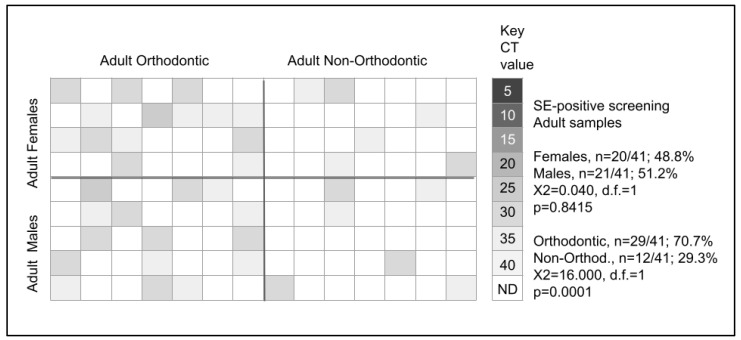
qPCR screening of adult samples for *Slackia exigua* (SE). Screening revealed *n* = 41/125 (32.8%) harbored *Slackia exigua* (SE), which was nearly equally divided among females (48.8%) and males (51.2%), *p* = 0.8415. The majority of *Slackia exigua* (SE)-positive samples were found among orthodontic patients (70.7%) versus non-orthodontic samples (29.3%), *p* = 0.0001.

Adult samples were also screened for the presence of *Slackia exigua* or SE (Figure 2). These data revealed that *n* = 41/125 or 32.8% of samples harbored *Slackia exigua* (SE). More detailed analysis of these data revealed that 20/41 or 48.7% of the *Slackia exigua* (SE)-positive samples were derived from adult females, with the remainder 21/41 or 51.3% from adult males, which was not statistically significant, *p* = 0.8415. However, analysis of these data by orthodontic status revealed that *n* = 29/41 or 70.7% of the *Slackia exigua* (SE)-positive samples were found among adult orthodontic patients with only *n* = 12/41 or 29.3% from adult non-orthodontic samples, which was statistically significant, *p* = 0.0001.

More detailed analysis of adult female orthodontic patients revealed *n* = 14/30 or 46.7% were *Slackia exigua* (SE)-positive compared with *n* = 6/32 or 18.8% of adult female non-orthodontic patients, which was much greater than expected (X2 = 40.960, d.f. = 1, *p* = 0.0001). Similarly, analysis of the adult male orthodontic samples revealed *n* = 15/30 or 50.0% were *Slackia exigua* (SE)-positive compared with *n* = 6/33 or 18.2% of adult male non-orthodontic patients, which was also greater than expected (X2 = 40.960, d.f. = 1, *p* = 0.0001).

To further evaluate these results by demographic variables, the qPCR screening data were compiled for further analysis (Table 3). The sorting of the *Slackia exigua* (SE)-positive and *Slackia exigua* (SE)-negative samples by Sex revealed no significant differences in the overall percentage of males (44%) and females (39.4%) that harbored this organism, *p* = 0.2301. In addition, within the pediatric samples, roughly equal percentages of pediatric male (53.5%) and pediatric female (45.7%) samples were found harboring *Slackia exigua* (SE), *p* = 0.4237. Similarly, nearly equal percentages of adult males (33.3%) and adult females (32.3%) were also found to be *Slackia exigua* (SE)-positive, *p* = 0.8633.

The analysis of these data by Age (adult or pediatric clinic population samples) demonstrated significant differences between *Slackia exigua* (SE)-positive samples. More specifically, a significantly higher percentage of pediatric samples (49.6%) harbored *Slackia exigua* (SE) compared to adult samples (32.8%), *p* = 0.0007. This age-specific relationship was also found with orthodontic status, with the percentage of *Slackia exigua* (SE)-positives among pediatric orthodontic patients (71.4%) much greater than among adult orthodontic patients ((48.3%), *p* = 0.001). This was also observed among the non-orthodontic samples with higher percentages of *Slackia exigua* (SE)-positives among pediatric non-orthodontic patients (28.2%) than adult non-orthodontic patients (18.4%), *p* = 0.0259.

The analysis of these data by Orthodontic status also revealed significant differences among these clinical patient samples. Overall, a larger percentage of *Slackia exigua* (SE)-positive samples were derived from orthodontic patients (60.8%) than non-orthodontic patients (23.5%), *p* = 0.0001. This did not differ significantly between male orthodontic patients (64.6%) and female orthodontic patients (59.6%), *p* = 0.1061, or between male non-orthodontic patients (24.6%) and female non-orthodontic patients (22.4%), *p* = 0.4689.

## 4. Discussion

The primary objective of this study was to evaluate the presence of *Slackia exigua* among clinical saliva samples from both adult and pediatric, as well as orthodontic and non-orthodontic clinic patients. These results clearly demonstrate the presence of this organism, which may be more highly concentrated among pediatric than adult patients. Although some previous studies have identified *Slackia exigua* among patients with early childhood caries, this may be the first study to evaluate this organism among a large cohort of pediatric and adult patients without pre-identifying these patients using specific disease associations, such as severe early childhood caries (SECC) or atypical periodontitis [7,11].

In addition, this study also found a strong association between orthodontic brackets and the prevalence of this organism, which appears to be most closely associated with pediatric orthodontic patients. To date, this may be the only study that has evaluated this potential relationship between the presence of orthodontic brackets and the oral prevalence of *Slackia exigua*. Although one previous study found some association between patients with dry sockets and the microbial prevalence of *Slackia exigua*, no information regarding age or orthodontic status was available to make additional inferences [18].

These data and the novel associations found within pediatric and orthodontic patient samples are important as new data continue to find associations between the presence of *Slackia exigua* and oral diseases, including periodontal disease and peri-implantitis [19]. Moreover, several studies have found that *Slackia exigua* may be involved in cases of bacteremia and septic shock, which involve other chronic illnesses and co-morbidities [20,21,22]. A more thorough understanding of which patients may harbor this organism and what factors might increase the oral prevalence (such as the presence of orthodontic brackets), would enable researchers to more clearly understand the risk potential associated with oral *Slackia exigua*.

Other studies from this group have analyzed the changes in oral microbial prevalence and changes to specific microbial populations, including *Scardovia wiggsiae*, *Selenomonas noxia*, *Streptococcus mutans*, and *Porphyomonas gingivalis*, which may be elevated among orthodontic patient populations in particular [23,24,25]. In addition, another study from this group recently found that the commensal bacterium *Akkermansia muciniphila* (which also exhibits anti-inflammatory properties) is decreased among adults versus pediatric patients, as well as among orthodontic versus non-orthodontic patients regardless of age within this clinic population [26]. In contrast, the results from the current study appear to suggest that the oral prevalence of *Slackia exigua* may be higher among orthodontic patients than non-orthodontic patients, which holds true regardless of age. However, although many oral pathogens may be typically more prevalent among adult patients, especially with orthodontic appliances, this study demonstrated that the highest percentages were found mainly among pediatric patients with fixed orthodontic appliances [27,28]. This may suggest some associations that may be age- or diet-related, although more research will be needed to determine which factors may contribute to these differences in oral prevalence.

There are some limitations associated with this study, which should also be considered when evaluating these results. Most importantly, this was a retrospective study of previously collected clinical samples from an existing biorepository. As patients were originally recruited based upon voluntary participation, some bias and differences may exist between patients who agreed to participate versus those who declined participation [23,24,25]. Furthermore, the recruitment of patients was performed exclusively within the clinical patient population at a public university-based dental school, which has been demonstrated to serve a large proportion of low-income and minority patients with public assistance programs including Medicaid—which may have significantly influenced the number of patients with poor oral health [29,30]. Finally, due to the specific limitations of the original protocols for the collection of saliva samples, several other confounding factors and variables regarding the oral health and clinical status of these patients remain unknown, such as the periodontal pocket depth (PPD), the decayed, missing, and filled teeth or DMFT score, and the overall caries risk of these patients. In addition, any improvements in orthodontic treatment protocols or biomaterials that might reduce bacterial adhesion or colonization should be noted for further evaluation and study. These data could be included in future prospective studies of this organism that will likely follow, which could be valuable information to include in future prospective studies of *Slackia exigua* [31,32,33].

## 5. Conclusions

This study provides evidence regarding the oral prevalence of *Slackia exigua*, which may be higher among pediatric patients compared with adults within this specific clinic population. In addition, this organism may be more prevalent among those with fixed orthodontic appliances regardless of their age—which may suggest that future studies may be needed to properly evaluate and monitor the associations of this organism with the onset and development of orthodontic-associated diseases, such as caries lesions, gingivitis, periodontal inflammation, and periodontal disease. Finally, due to the ability for this anaerobic organism to survive within the gastrointestinal tract, this may be among the first clinical studies to document these younger populations who may be in the early stages of oral infection that may lead to seeding and colonizing the gut microbiome of these patients, which is important for understanding the microbial ecology and the development of organisms within various tissues and organ systems over time.

## Figures and Tables

**Table 1:** Demographic analysis of study sample.

Demographic	Study Sample	Clinic Population	Statistical Analysis
*Sex*			
Female	*n* = 132/266 (49.6%)	49.1%	X2 = 0.100, d.f. = 1
Male	*n* = 134/266 (50.4%)	50.9%	*p* = 0.7518
*Race/Ethnicity*			
White	*n* = 93/266 (34.9%)	34.6%	X2 = 0.040, d.f. = 1
Minority	*n* = 173/266 (65.1%)	65.4%	*p* = 0.8422
Hispanic	*n* = 151/266 (56.7%)	58.6%	
*Age*			
Pediatric *n* = 141	13.1 years (Average) 7–17 years (Range)	10.4 years (Average) 0–17 years (Range)	Two-tailed t-test *p* = 0.021
Adult *n* = 125	40.6 years (Average) 18–77 years (Range)	42.3 years (Average) 18–89 years (Range)	Two-tailed t-test *p* = 0.038

**Table 2:** DNA isolation and analysis.

Study Samples	DNA Concentration [ng/uL]	DNA Purity A260:A280 Ratio
Pediatric patient samples	Average: 481.2 ng/uL +/− 55.1 Range: 127.2–769.1 ng/uL	Average: 1.73 Range: 1.69–1.82
Adult patient samples	Average: 455.3 ng/uL +/− 61.2 Range: 189.5–776.2 ng/uL	Average: 1.75 Range: 1.66–1.85

**Table 3:** Analysis of *Slackia exigua* (SE)-positive and *Slackia exigua* (SE)-negative samples by Sex, Age, and Orthodontic status.

Demographic	*Slackia exigua* (SE)-Positive	*Slackia exigua* (SE)-Negative	Statistical Analysis
*Sex*			
Male	*n* = 59/134 (44.0%)	*n* = 75/134 (56.0%)	X2 = 0.1440, d.f. = 1
Female	*n* = 52/132 (39.4%)	*n* = 80/132 (60.6%)	*p* = 0.2301
Pediatric Male	*n* = 38/71 (53.5%)	*n* = 33/71 (46.5%)	X2 = 0.640, d.f. = 1
Pediatric Female	*n* = 32/70 (45.7%)	*n* = 38/71 (53.5%)	*p* = 0.4237
Adult Male	*n* = 21/63 (33.3%)	*n* = 42/63 (66.7%)	X2 = 0.030, d.f. = 1
Adult Female	*n* = 20/62 (32.3%)	*n* = 42/62 (67.7%)	*p* = 0.8633
*Age*			
Adult	*n* = 41/125 (32.8%)	*n* = 84/125 (67.2%)	X2 = 11.560, d.f. = 1
Pediatric	*n* = 70/141 (49.6%)	*n* = 71/141 (50.4%)	*p* = 0.0007
Adult Orthodontic	*n* = 29/60 (48.3%)	*n* = 31/60 (51.7%)	X2 = 16.006, d.f. = 1
Pediatric Orthodontic	*n* = 50/70 (71.4%)	*n* = 20/70 (28.6%)	*p* = 0.001
Adult Non-Orthodontic	*n* = 12/65 (18.4%)	*n* = 53/65 (81.6%)	X2 = 4.960, d.f. = 1
Pediatric Non-Orthodontic	*n* = 20/71 (28.2%)	*n* = 51/71 (71.8%)	*p* = 0.0259
*Orthodontic status*			
Orthodontic	*n* = 79/130 (60.8%)	*n* = 51/130 (39.2%)	X2 = 54.000, d.f. = 1
Non-orthodontic	*n* = 32/136 (23.5%)	*n* = 104/136 (76.5%)	*p* = 0.0001
Male Orthodontic	*n* = 42/65 (64.6%)	*n* = 23/65 (35.4%)	X2 = 2.611, d.f. = 1
Female Orthodontic	*n* = 37/65 (56.9%)	*n* = 28/65 (43.1%)	*p* = 0.1061
Male Non-Orthodontic	*n* = 17/69 (24.6%)	*n* = 52/69 (75.4%)	X2 = 0.524, d.f. = 1
Female Non-Orthodontic	*n* = 15/67 (22.4%)	*n* = 52/67 (77.6%)	*p* = 0.4689

## Data Availability

The data presented in this study are available on request from the corresponding author. The data are not publicly available due to the study protocol data protection parameters requested by the IRB and OPRS for the initial study approval.
